# Pre-hospital severe traumatic brain injury – comparison of outcome in paramedic versus physician staffed emergency medical services

**DOI:** 10.1186/s13049-016-0256-x

**Published:** 2016-04-29

**Authors:** Toni Pakkanen, Ilkka Virkkunen, Antti Kämäräinen, Heini Huhtala, Tom Silfvast, Janne Virta, Tarja Randell, Arvi Yli-Hankala

**Affiliations:** FinnHEMS Ltd, Research and Development Unit, Vantaa, Finland; Department of Anaesthesia, Tampere University Hospital, Tampere, Finland; Tays Emergency Medical Service, FinnHEMS 30, Tampere University Hospital, Tampere, Finland; School of Health Sciences, University of Tampere, Tampere, Finland; Department of Anaesthesia and Intensive Care, Helsinki University Hospital, University of Helsinki, Helsinki, Finland; Medical School, University of Tampere, Tampere, Finland

**Keywords:** Pre-hospital Emergency Care (MeSH), Emergency Medical Services (MeSH), Critical Care (MeSH), Traumatic Brain Injury (MeSH), Airway Management (MeSH), Endotracheal Intubation (MeSH), Patient Outcome Assessment (MeSH), Glasgow Outcome Scale (MeSH)

## Abstract

**Background:**

Traumatic brain injury (TBI) is one of the leading causes of death and permanent disability. Emergency Medical Services (EMS) personnel are often the first healthcare providers attending patients with TBI. The level of available care varies, which may have an impact on the patient’s outcome. The aim of this study was to evaluate mortality and neurological outcome of TBI patients in two regions with differently structured EMS systems.

**Methods:**

A 6-year period (2005 – 2010) observational data on pre-hospital TBI management in paramedic-staffed EMS and physician-staffed EMS systems were retrospectively analysed. Inclusion criteria for the study were severe isolated TBI presenting with unconsciousness defined as Glasgow coma scale (GCS) score ≤ 8 occurring either on-scene, during transportation or verified by an on-call neurosurgeon at admission to the hospital. For assessment of one-year neurological outcome, a modified Glasgow Outcome Score (GOS) was used.

**Results:**

During the 6-year study period a total of 458 patients met the inclusion criteria. One-year mortality was higher in the paramedic-staffed EMS group: 57 % vs. 42 %. Also good neurological outcome was less common in patients treated in the paramedic-staffed EMS group.

**Discussion:**

We found no significant difference between the study groups when considering the secondary brain injury associated vital signs on-scene. Also on arrival to ED, the proportion of hypotensive patients was similar in both groups. However, hypoxia was common in the patients treated by the paramedic-staffed EMS on arrival to the ED, while in the physician-staffed EMS almost none of the patients were hypoxic. Pre-hospital intubation by EMS physicians probably explains this finding.

**Conclusion:**

The results suggest to an outcome benefit from physician-staffed EMS treating TBI patients.

**Trial registration:**

ClinicalTrials.gov ID NCT01454648

## Background

Worldwide, traumatic brain injury (TBI) is one of the leading causes of death and permanent disability [1] particularly in young adults. After the initial injury, many patients suffer secondary brain injuries because of hypoxia, hypercapnea and hypotension. The secondary brain injuries can result in increased mortality and disability [1].

The management of severe TBI focuses on the prevention of secondary ischemic brain injury by optimizing the balance between cerebral oxygen delivery and utilization [2, 3]. Cerebral oxygen delivery is partly determined by the arterial oxygen content and partly by cerebral blood flow (CBF), and therefore is affected by cerebral autoregulation. When the autoregulation is impaired, a correlation between mean arterial pressure (MAP) and CBF exists, making the brain susceptible to ischemia or hyperemia [4, 5].

Half of those who die from TBI do so within the first two hours of injury [1]. Emergency Medical Services (EMS) personnel are often the first healthcare providers attending patients with TBI [1]. Thus, pre-hospital assessment and treatment is a critical link in providing appropriate care [6] as the prognosis of patients with severe TBI and low Glasgow Coma Scale (GCS) score depends strongly on early support of vital functions [3, 7]. In particular, pre-hospital prevention of hypoxia by adequate respiratory management including secured airway, normoventilation and prevention of aspiration is strongly associated with improved outcome [8–11]. Depending on the structure of the EMS system, the level of available care varies, which may have an impact on the patient’s outcome.

The aim of this study was to evaluate mortality and neurological outcome of TBI patients in two regions with differently structured EMS systems.

## Methods

### Description of the EMS system

Finland covers an area of 337,000 km2 with a population of 5.4 million. Half of the population lives in the south, whereas the middle and especially northern parts of the country are rural. In larger cities, the fire brigade is the usual EMS provider, whereas private entrepreneurs are most frequent in rural areas. The EMS system in general is three-tiered: basic life support (BLS), advanced life support (ALS) and physician-staffed units. Rescue department fire engines can also be used as first responders. BLS units are usually manned with fire fighters and authorized to use for example an automated external defibrillator (AED), perform tracheal intubation of a lifeless adult patient and to establish an intravenous line. The advanced level employs nurses and paramedics with 3.5 – 4 years of training who are authorized e.g. to give intravenous drugs, provide sedation to facilitate tracheal intubation in unconscious patients and initiate thrombolytic treatment after consulting with a physician. In cities, response times for basic units average 5–7 min, ALS response times vary between 10 and 15 min. Physician-staffed ground vehicles are used in two cities and five helicopter based physician-staffed units cover other parts of the country. The physician-staffed unit calls are not restricted to trauma as they respond to medical emergencies as well.

### The Pirkanmaa area paramedic-staffed EMS system (EMS)

The Pirkanmaa Hospital District is situated in Western Finland. There are approximately 200 000 inhabitants living in the city of Tampere and another 250 000 inhabitants in the surrounding communities. At the time of the study, there were no dedicated medical directors, and EMS crews consulted on-call hospital physicians and local general practitioners for treatment guidelines. Physician-staffed pre-hospital units and on-line medical supervision were not available. Paramedic-staffed EMS units provided pre-hospital care in this region. During the study period, patients with a decreased level of consciousness were routinely administered oxygen according to national guidelines. Neuromuscular blocking agents were not available in the pre-hospital service and pre-hospital advanced airway management was performed using sedatives and opioids only.

### The Helsinki and Uusimaa area physician-staffed EMS system (Ph-EMS)

The Helsinki and Uusimaa Hospital District is situated in the southern part of Finland with a total of 1.3 million inhabitants living in the capital area. During the study period, the hospital district’s EMS system was a three-tiered system with two physician-staffed units: a physician-staffed mobile intensive care unit (MICU) and a Helicopter Emergency Medical Service (HEMS) unit providing the third tier. Patients with a decreased level of consciousness were routinely administered oxygen according to national guidelines. The physician-staffed EMS units were dispatched on primary missions together with basic or advanced life support EMS units to patients with potential major trauma or critical medical conditions. The physicians are dedicated anaesthesiologists with extensive experience in pre-hospital emergency medicine. In the physician-staffed units, general anaesthesia including neuromuscular blocking agents could be used to facilitate rapid sequence intubation (RSI).

### Neurosurgical care

Within both study areas, a university hospital operated as the referral centre providing standardized immediate neurosurgical care according to national guidelines (the first edition published in 2003, with an update in 2008). Both facilities operate according to similar treatment principles, in terms of criteria for surgical interventions and timing of surgery.

### Study design

A 6-year period (2005–2010) observational data on pre-hospital severe TBI management in both EMS systems were retrospectively analysed. Patients included in the study were identified from the university hospital patient records based on the ICD-10 discharge diagnoses for traumatic brain injury or for skull fracture (S06.2-S06.6, S06.8, S02.1). Inclusion criteria for the study were severe isolated TBI presenting with unconsciousness defined as Glasgow coma scale (GCS) score ≤8 [12] occurring either on-scene, during transportation or verified by an on-call neurosurgeon at admission to the hospital. Patients with concomitant multiple injuries with the need for surgical interventions (other than neurosurgery) were excluded, as were patients transferred from other hospitals (secondary transfers). Desaturation was defined as a decrease in SpO_2_ to below 90 %. Hypotension was defined as a decrease in systolic blood pressure (SBP) below 90 mmHg. These definitions are consistent with the latest edition of the Brain Trauma Foundation’s guidelines for pre-hospital management of traumatic brain injury [1].

Age, gender, EMS response and total mission time, airway related variables, mechanism of injury, GCS score and vital signs at the scene and on arrival to the emergency department (ED) were reviewed and cross-referenced with EMS run-sheets and ED documentation. Outcome evaluation was performed based on hospital patient records one year after the incident. In multivariable analysis, hypoxia and hypotension were used as risk factors for mortality based on previous studies [3, 7] as well as age and GCS score based on them being among the core variables in the IMPACT [13] and CRASH [14] prognostic TBI models.

For assessment of neurological outcome, a modified Glasgow Outcome Score (GOS) was used [15]. A GOS of 1 denoted death within a year, GOS 2–3 poor neurological outcome (need for assistance in activities of daily life) and GOS 4–5 corresponded to good neurological recovery (independent life). The outcome evaluation was performed by one of the authors (T.P.) based on hospital patient records six months after the incident. If the evaluation was unclear, the research team members reviewed the case and a joint decision was made. Data on the time of death were obtained from the national statistical authority Statistics Finland.

The study was approved by Regional Ethics Committee of the Pirkanmaa Hospital District (R09161), permission to conduct the study was obtained from the Research Directors of Tampere and Helsinki University Hospitals and the study was registered in ClinicalTrials.gov (Identifier NCT01454648).

### Statistical analyses

Results are expressed as medians and ranges or percentages. EMS groups were compared using chi-square or Fishers exact test for categorical variables. The odds ratios and 95 % confidence intervals were calculated using univariate and multivariable binary logistic regression to identify predictors of good neurological outcome and one-year mortality. The one-year survival was characterized using Kaplan-Meier plot and the log-rank test was used to compare groups. Statistical significance was considered at a value of less than 0.05. The data were analysed using IBM SPSS Statistics for Windows Version 21.0. Armonk, NY: IBM Corp. Released 2012.

## Results

**Fig. 1:**
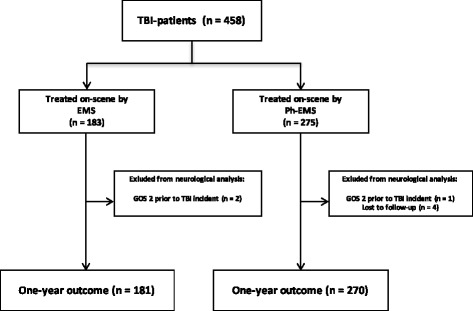
Flowchart

During the 6-year study period a total of 458 patients met the inclusion criteria (Fig. 1). The complete data of 181 patients in the EMS and 270 patients in the Ph-EMS were available for final neurological outcome analysis. The baseline characteristics are presented in Table 1.

**Table 1:** Baseline characteristics

	EMS		Ph-EMS		
	*n* = 183		*n* = 275		
	mean	SD, range	mean	SD, range	*p*-value
Age, years	52	21.6, 6–89	47	19.7, 0.2–90	0.014
	n	%	n	%	
Male	127	69	204	74	0.263
Mechanism of injury					0.018
Fall from ground level	81	44	105	38	
Traffic accident	41	22	51	19	
Fall from a height (>2 m)	25	14	36	13	
Violence	14	8	24	9	
Other	4	2	25	9	
Unknown	18	8	34	12	
Primary GCS					
Median	4	-	4	-	0.370
≤8	137	75	247	90	
9–13	13	7	14	5	
14–15	6	3	7	3	
Unknown	27	15	7	3	

The time from dispatch to the arrival of the first EMS unit on-scene did not differ between the groups: the median response time was 8 (range 0–37) minutes in the EMS and 9 (range 0–62) minutes in the Ph-EMS groups (*p* = 0.246). However, the total mission times (from dispatch to arrival to ED) were shorter in the EMS group: median time of 54 (range 18–180) minutes compared to Ph-EMS group 72 (range 23–191) minutes (*p* <0.001).

First recorded systolic blood pressure on-scene was hypotensive (<90 mmHg) in 4 % in the EMS treated group, and hypoxia (SpO_2_ <90 %) was documented in 19 % of the patients. The corresponding figures in the Ph-EMS treated patients were 3 % (*p* = 0.44) and 15 %, (*p* = 0.31), respectively. Advanced airway management was performed in 16 % of the patients in the EMS group and in 98 % of the patients in the Ph-EMS group (*p* <0.001). Details on airway management are described in Table 2. On arrival to ED, hypotension was recorded in 4 % in both study groups but the patients in the EMS group were more often hypoxic (10 % vs. 1 %, OR 10.05 CI 2.91–34.67, *p* <0.001). Outcome by secondary insult at the time of arrival at ED is presented in Table 3.

**Table 2:** Pre-hospital airway management

	EMS		Ph-EMS		
	*n*	%	*n*	%	*p*-value
Airway secured	29	16	269	98	<0.001
Intubation (drug-facilitated)	19	10	263	96	
Intubation (without medication)	6	3	0	0	
Supraglottic device	4	2	5	2	
Surgical airway	0	0	1	0	
Not secured	154	84	6	2	
Failed pre-hospital intubation attempt(s)	3	2	0	0

**Table 3:** Outcome by secondary insult at the time of arrival at ED

	EMS					Ph-EMS				
Secondary			Outcome					Outcome		
Insult	*n*	%	Good	Poor	Dead	*n*	%	Good	Poor	Dead
Neither	149	86.6	33.6 %	10.7 %	55.7 %	246	94.6	38.6 %	17.5 %	43.9 %
Hypoxia	17	9.9	23.5 %	17.6 %	58.8 %	4	1.5	25.0 %	0 %	75.0 %
Hypotension	5	2.9	40.0 %	20.0 %	40.0 %	10	3.9	10.0 %	40.0 %	50.0 %
Both	1	0.6	0 %	0 %	100 %	0	0	0 %	0 %	0 %
Total	172	100	32.6 %	11.6 %	55.8 %	260	100	37.3 %	18.1 %	44.6 %
Data not available for 9 patients.						Data not available for 10 patients.				

**Fig. 2:**
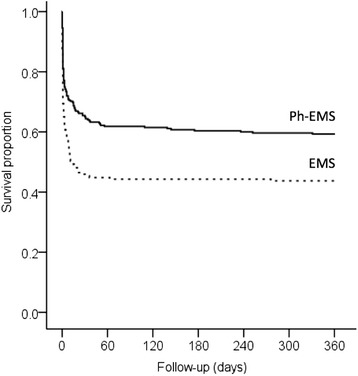
One-year survival in relation to EMS system

One-year mortality was higher in the EMS group: 57 % vs. 42 % (OR 1.86 CI 1.27–2.71, *p* = 0.001). Good neurological outcome was less common in patients treated in the EMS group: 32 % of the EMS and 38 % (OR 0.74 CI 0.5–1.11, *p* = 0.14) of the Ph-EMS treated patients had a good neurological recovery (GOS 4–5) with independent life one year after the event. In the multivariable analysis after the patients were adjusted by age (OR 1.05 CI 1.04–1.07, *p* <0.001), the EMS-system remained as a significant risk factor for mortality (OR 1.69 CI 1.11–2.58, *p* = 0.015). Long-term mortality of the two patient groups is illustrated in Fig. 2.

## Discussion

In this observational retrospective study the results point to outcome benefit from physician-staffed EMS treating TBI patients. Mortality was significantly lower and neurological outcome better in patients in the physician-staffed EMS group compared to the paramedic-staffed EMS group.

Pre-hospital advanced airway management of TBI patients is well defined in international guidelines: an airway should be established in patients who have severe TBI (GCS ≤8), have the inability to maintain an adequate airway or are hypoxemic, which is not corrected by supplemental oxygen by the most appropriate means available [1]. In the pre-hospital setting endotracheal intubation has potential advantages: oxygenation can be optimised and controlled ventilation is possible with the airway secured. The optimal way of securing the airway still remains controversial [16, 17]. If RSI is performed poorly, hypoxia and hypotension have been shown to have a negative effect on outcome of TBI patients undergoing pre-hospital RSI [17].

In this study, the airway was secured in the pre-hospital setting in almost all of the patients in the physician-staffed EMS group and only in few patients in the paramedic-staffed EMS group. Anaesthetics were available for the EMS physicians, while the paramedics were limited to the use of sedatives only, which might have an effect on the rate of airway management procedures.

In earlier studies both hypoxemia and hypotension have been shown to have a negative impact on outcome [3, 8]. Desaturations (SpO_2_ <70 %) during intubation or any oxygen desaturation (SpO_2_ <90 %) has been associated with higher mortality [17]. The incidence of hypotension in patients with TBI upon first contact in the field has been reported to be between 16–19 % [18, 19]. A single episode of hypotension has been associated with increased mortality when compared with a matched group of patients without hypotension [3].

We found no significant difference between the study groups when considering the secondary brain injury associated vital signs on-scene. Also on arrival to ED, the proportion of hypotensive patients was similar in both groups. However, hypoxia was common in the patients treated by the paramedic-staffed EMS on arrival to the ED, while in the physician-staffed EMS almost none of the patients were hypoxic. Pre-hospital intubation by EMS physicians probably explains this finding. Detailed data on vital signs covering the whole pre-hospital phase in the study groups were not available in this retrospective study, so the presence of momentary hypoxia or hypotension during the pre-hospital period could not be further evaluated.

Due to the low rate of intubation in the paramedic-staffed EMS group, ventilatory parameters could not be compared. Arterial blood gas results from the ED were documented in 85 % of the physician EMS group and in 48 % of the paramedic EMS group. When analysing this further we found that only 36 % of the arterial blood gas samples in the physician EMS group and 14 % in the paramedic EMS group were analysed within 10 min or less after arrival to the ED and would in our opinion represent the oxygenation and ventilation during the pre-hospital phase. Therefore no further analysis was made.

There were no differences between the groups in gender, mechanism of injury, EMS response times or initial GCS. When the patient groups were adjusted by age, the EMS-system still remained as a significant variable in multivariable regression analysis of mortality risk factors.

The finding that the physician EMS group produced more patients with poor neurological outcome, can possibly be explained by Stoccheti’s hypothesis: “The quality of the overall trauma system affects the outcome of the series because a better trauma system produces less favourable outcomes. This apparent paradox is due to the fact that a more efficient trauma system brings even the most severe cases to the hospital” [20].

### Study limitations

This was an observational retrospective study and some limitations should be considered when interpreting the results. The pre-hospital data were originally self-reported and could not be independently verified and can therefore be biased. When considering the age distribution, the groups were not originally identical. Reliable pupil assessment was not recorded on all of the patients. Complete data on vital signs covering the pre-hospital phase were not available for all patients. The outcome evaluation was based on patient record assessment without physical examination or the help of a questionnaire. The first CT scans were not evaluated using the Marshall classification. It is possible that the deaths occurring at the late phases of the follow-up period were unrelated to the pre-hospital index situations and secondary diseases or injuries could have influenced patient survival and outcome during the follow-up period.

## Conclusions

Based on available data, the results suggest to an outcome benefit from physician-staffed EMS treating TBI patients. Further prospective multicentre studies with more thoroughly data of vital signs covering the pre-hospital phase, total pre-hospital treatment and the outcome evaluation are needed to confirm the hypothesis.

## Abbreviations

ED: emergency department
EMS: emergency medical services
GCS: Glasgow coma scale
GOS: Glasgow outcome score
HEMS: helicopter emergency medical service
MICU: mobile intensive care unit
RSI: rapid sequence intubation
TBI: traumatic brain injury
